# Mechanisms of Plant Antioxidants Action

**DOI:** 10.3390/plants10010035

**Published:** 2020-12-25

**Authors:** Davide Barreca

**Affiliations:** Department of Chemical, Biological, Pharmaceutical and Environmental Sciences, University of Messina, 98168 Messina, Italy; dbarreca@unime.it

**Keywords:** antioxidants, polyphenols, free radicals, carotenoids, vitamins, flavonoids, activation/block of the signal cascade, antioxidant modulation of key metabolic enzymes, oxidative stress, hydrogen atom transfer, single electron transfer, antioxidant assays

## Abstract

The plant kingdom is a rich source of health-promoting compounds and has always played a fundamental role in the isolation, identification, and modification of compounds able to perform several properties on live organisms. Among them, the so-called “antioxidants” have a major potentiality to increase human wellness. Antioxidants are important components in the signaling and defense mechanisms in some plants, where they are precursors of compounds of greater complexity, the modulator of plant growth, and the defensive system against pathogenic organisms and predators. The extraordinary variety of chemical structure and substitution present in the different plant antioxidants make them an inestimable source of interesting compounds, with the ability to counter reactive oxygen/nitrogen species (ROS/RNS) and to stimulate the activation of signal cascade inside the cells. The mechanisms by which antioxidants detoxify these dangerous compounds are complex and involve either direct or indirect interaction with radicals. Antioxidants inhibit or quench free radical reactions mainly based on their reducing capacity or hydrogen atom-donating capacity, their solubility, and chelating properties. Moreover, their ability to modulate key metabolic enzymes and activate/block gene transcription also has remarkable importance.

## Introduction

Taking into account the high interest in plant antioxidants as an emerging challenge for the development of health-promoting compounds, crop protection, and potential biotechnological applications [1,2,3,4,5,6], I am very pleased to introduce this Special Issue, which aims to cover a wide variety of areas, aiming to contribute to the overall knowledge of the molecular mechanism of antioxidant action by multiple points of view on both the producer and the consumer organisms. This issue is dedicated to Prof. Guddadarangavvanahally K. Jayaprakasha (Vegetable and Fruit Improvement Center, Texas A&M University), who has significantly contributed to this Special Issue and unfortunately left us during this year. He embodied humility and empathy and was an inspiration with his dedication to science and ethics to students and colleagues. I remembered him with scientific esteem for his dedication to advancing science and I was honored by his friendship and he was an amazing person, always ready to help anyone in need. Dr. Jay was an unselfish, dedicated, and passionate scientist and made a lasting impact on several students and scientists around the world, according to his supervisor, Dr. Bhimu Patil (Director of Vegetable and Fruit Improvement Center, Texas A&M University). The long-term impact of his research will save lives and forever leave a footprint on the earth. One of the best examples of his impactful research is based on the isolation and characterization of unique and novel health-promoting compounds from fruits and vegetables, in particular *Citrus* and turmeric. This research would advance science to the next level.

I have received many excellent manuscripts that try to shed some light on the potential mechanism of action of antioxidants and also described the identification of new compounds, bringing to the collection nine articles [7,8,9,10,11,12,13,14,15] and two reviews [16,17].

Never, in this hard year, has the necessity to develop alternative solutions and strategies to fight microorganisms and viruses been so timely. Herpes simplex virus (HSV) infection is very common in humans and is associated with oral mucocutaneous lesions and/or genital infections. In the article entitled “In Vitro Anti-HSV-1 Activity of Polyphenol-Rich Extracts and Pure Polyphenol Compounds Derived from Pistachios Kernels (*Pistacia vera* L.)”, the authors [7] have reported on the activity of polyphenol-rich extracts of pistachio kernels on HSV-1 replication. The results obtained showed that pistachio polyphenols have remarkable inhibitory activity against HSV-1. Natural compounds could become great candidates for the development of novel topical or oral drug formulations to treat HSV-1 infections, either alone or in combination with standard antiviral therapies.

Three works (two articles and one review) focused on the properties of melatonin. Melatonin is an interesting molecule with complex functions and potentiality [10,15,16]. Khan et al. [16] have analyzed the mechanism of exogenous melatonin in various crops that can improve plant growth and development in response to various abiotic and biotic stresses by regulating antioxidant machinery in plants (both the enzymatic and non-enzymatic antioxidant defense systems), with a concomitant decrease in lipid peroxidation and plasma membrane damage. They highlight that some aspects have been elucidated, but a detailed transcriptomic analysis is required to reveal the gene regulatory networks involved in the effects of exogenous melatonin application.

Ibrahim et al. [15], starting from the recent observation that melatonin can be considered a growth regulator with multiple physiological functions in plants and its exogenous application can decrease the deleterious effects of drought stress, have analyzed foliar application of melatonin to tomatoes under long-term optimal and deficient irrigation conditions to improve crop productivity and quality. In the range of 0–40 ppm, melatonin increases plant growth, total fruit yield, quality attributes (soluble solids, ascorbic acid, and lycopene content), chlorophyll content, and antioxidant enzyme activities (such as ascorbate peroxidase, catalase, and peroxidase), with a concomitant reduction of the amino acid proline, soluble sugars, H_2_O_2_, and dangerous substances (such as malondialdehyde) derived from oxidative injuries to cellular components, suggesting the possible utilization of melatonin under specific conditions and in enhancing tomato tolerance to deficient irrigation. The stress tolerance topic has also been analyzed by Buttar et al. [15]. The authors have analyzed the protection mechanism of melatonin on wheat seedlings treated with heat stress. Their results show the involvement of melatonin in modulating the antioxidant defense system, with the activation of the ascorbate–glutathione cycle, the stabilization of photosynthetic machinery by increasing the chlorophyll amount, and the induction of the expression of reactive oxygen species (ROS)-related genes, TaSOD, TaPOD, and TaCAT, and anti-stress responsive genes, such as TaMYB80, TaWRKY26, and TaWRKY39.

Laganà et al. [12] have described the anthocyanin profile of lemon bottlebrush (*Callistemon citrinus* (Curtis) Skeels) flowers by RP-HPLC-DAD-ESI-MS/MS analysis of an anthocyanin enriched fraction of acidified methanolic extract and performed a deep analysis of its antioxidant, cytoprotective, and anti-angiogenetic properties. The work led to the identification of four anthocyanins: cyanidin-3,5-*O*-diglucoside (cyanin), peonidin-3,5-*O*-diglucoside (peonin), cyanidin-3-*O*-glucoside, and cyanidin-coumaroylglucoside-pyruvic acid. The abilities of cultivation, robustness, and adaptability to different environments make the plant a useful source of anthocyanins, which show interesting applications due to their unique structural/chemical features. The anthocyanin-enriched fraction of acidified methanolic extract is able to actively scavenge DPPH, ABTS, and AAPH radicals and to counteract β-carotene bleaching. Based on these data, the effects of hydroxylation and methoxylation at the level of the B ring have a remarkable effect on antioxidant activity and the basic structure of anthocyanins. Moreover, they protect human mononuclear cells from oxidative injuries and prevent angiogenesis. 

Samaniego et al. [11] described the influence of the maturity stage on the phytochemical composition and the antioxidant activity of four Andean blackberry cultivars (*Rubus glaucus* Benth) from Ecuador. They noticed a significant variation, based on the maturity stage among the variety analyzed, in the antioxidant and nutritional properties of the fruits.

Omidpanah et al. [8] have analyzed the influences of monoterpenes of *Trachyspermum ammi* on the viability of spermatogonia stem cells. Real-time polymerase chain reaction was used to evaluate the expressions of several genes (promyelocytic leukemia zinc finger protein, DNA-binding protein inhibitor, tyrosine protein kinase, B-cell lymphoma 2, and Bcl2-associated X protein) after treatment with oil obtained by hydrodistillation. The results reveal a correlation with the amount of oil used, in particular with thymol, suggesting its role in the improved quality and viability of spermatogonia cells in cell culture and the activation/blocking of specific signals inside the cells.

Metrani et al. [14] have conducted a study to analyze the quality of functional food due to the added value of phytochemicals and the nutritional composition of red onions. The study reported an analysis of polyphenol, protein, nitrogen, mineral, sugar, pyruvate, antioxidant, and α-amylase inhibition activities of red onion cultivars, sweet Italian and honeysuckle, grown in California and Texas, respectively. Cyanidin-3-(6″-malonoylglucoside) and cyanidin-3-(6″-malonoyl-laminaribioside) were the major polyphenols present in the cultivars, as evident from the principal component analysis of different cultivars of red onion. Cyanidin derivatives possessed strong antioxidant activity and are responsible for the inhibition of α-amylase. 

Singh et al. [13] have performed a multivariate analysis of amino acids and health-benefiting properties of cantaloupe varieties grown in six locations in the United States as a dietary source of protein and amino acids. The variation in amino acid amount is an indicator for quality control, and the selection of melon varieties for a commercial purpose from different locations. They were found to differ remarkably in function regarding their origin. The differences are also relevant for the phenolic content and in the antioxidant potential. 

Lombardo et al. [9] analyzed the mechanisms underlying the anti-inflammatory activity of bergamot essential oil and its antinociceptive effects. The bergamot essential oil without furocoumarins showed remarkable antioxidant activity that may contribute to inhibiting carrageenan-induced paw edema in rat and it shows analgesic properties. This paper provides new insight on the mechanisms underlying the anti-inflammatory and analgesic activity of bergamot essential oil (BEO). Because of the well-known toxicity of furocoumarins, authors performed this study using the BEO fraction deprived of these compounds (BEO-FF). To study the BEO-FF mode of action, it was used the carrageenan-induced hind paw oedema, a suitable experimental model of acute inflammation. In this context, BEO-FF exerted a significant inhibition of paw oedema due to a reduction of interleukin (IL)-1, IL-6, and tumour necrosis factor (TNF)-α levels in the paw homogenates, as well as nitrite/nitrate and prostaglandin E2 (PGE2) content in exudates. In order to evaluate the anti-nociceptive activity of BEO-FF, and to distinguish between peripheral and central analgesic effects, authors carried out both writhing and hot plate tests in mice. Results of the former one showed that BEO-FF elicited a pronounced analgesic response, likely due to the inhibition of nociceptive mediators release, whereas the results of the latter one suggested that the supra-spinal analgesia participated in the anti-nociceptive effects of BEO-FF.

The review of Speranza et al. [17] provides a general botanical and ethnobotanical overview summarizing up-to-date knowledge on the phytochemistry and biological properties of *Isatis tinctoria* L. (Brassicaceae), a medicinal plant commonly known as woad, and also historically used as an indigo dye. Currently, *I. tinctoria* is commonly used as a medicinal remedy and as a cosmetic ingredient. In 2011, a monograph of *I. tinctoria* was officially included in the European Pharmacopoeia. *Isatidis radix* is a well-known traditional Chinese medicine (TCM). Most recently, the National Health of Commission of P.R. China proposed *Isatidis radix* granula as a treatment against COVID-19. Many studies have shown anti-inflammatory, anti-tumor, antimicrobial, antiviral, analgesic, and antioxidant activities. The phytochemical composition of *I. tinctoria* has been thoroughly updated in the review, including information on the attempts in plant biotechnology studies at the enhanced production of flavonoids and alkaloids from *I. tinctoria* hairy root and shoot cultures as an alternative to plant raw materials.
